# Experimental Investigation of Cobalt Deposition on 304 Stainless Steel in Borated and Lithiated High-Temperature Water

**DOI:** 10.3390/ma16103834

**Published:** 2023-05-19

**Authors:** Jian Deng, Jieheng Lei, Guolong Wang, Lin Zhong, Mu Zhao, Zeyong Lei

**Affiliations:** 1 School of Nuclear Science and Technology, University of South China, Hengyang 421001, China; 2015002047@usc.edu.cn (J.D.); 2017000021@usc.edu.cn (L.Z.); 2 School of Mechanical Engineering, University of South China, Hengyang 421001, China; wgl18008453785@sina.com; 3 School of Electrical Engineering, University of South China, Hengyang 421001, China; 2016000038@usc.edu.cn; 4 China Nuclear Industry 24 Construction Co., Ltd., Beijing 102400, China; velvet010907@163.com

**Keywords:** 304SS, cobalt, deposition, spinel, primary loop

## Abstract

The radioactive corrosion products ^58^Co and ^60^Co in the primary loops of pressurized water reactors (PWRs) are the main sources of radiation doses to which workers in nuclear power plants are exposed. To understand cobalt deposition on 304 stainless steel (304SS), which is the main structural material used in the primary loop, the microstructural characteristics and chemical composition of a 304SS surface layer immersed for 240 h in borated and lithiated high-temperature water containing cobalt were investigated with scanning electron microscopy (SEM), X-ray diffraction (XRD), laser Raman spectroscopy (LRS), X-ray photoelectron spectroscopy (XPS), glow discharge optical emission spectrometry (GD-OES), and inductively coupled plasma emission mass spectrometry (ICP-MS). The results showed that two distinct cobalt deposition layers (an outer layer of CoFe_2_O_4_ and an inner layer of CoCr_2_O_4_) were formed on the 304SS after 240 h of immersion. Further research showed that CoFe_2_O_4_ was formed on the metal surface by coprecipitation of the iron preferentially dissolved from the 304SS surface with cobalt ions from the solution. The CoCr_2_O_4_ was formed by ion exchange between the cobalt ions entering the metal inner oxide layer and (Fe, Ni) Cr_2_O_4_. These results are useful in understanding cobalt deposition on 304SS and have a certain reference value for exploring the deposition behavior and mechanism of radionuclide cobalt on 304SS in the PWR primary loop water environment.

## 1. Introduction

Radiation protection is one of the most important issues in the operation of nuclear power plants. In fact, nonradioactive corrosion or abrasion products on the surfaces of metal components in the primary loop system of a PWR nuclear power plant are activated by neutrons in the core and form radioactive corrosion products (ACPs) (^51^Cr, ^54^Mn, ^58^Co, ^59^Fe, ^60^Co, ^110m^Ag, ^122^Sb), which are transferred outside the core by the coolant and deposited on the inner surfaces of the equipment, forming radiation fields [1,2]. Among them, ^60^Co and ^58^Co are the main radioactive products formed in the primary loop and the main sources of irradiation doses for power plant maintenance personnel; approximately 90% or more of the collective dose comes from the deposited corrosion products [3,4,5]. Therefore, it is important to explore the deposition behaviors of ACPs on the metal surfaces of PWRs under normal conditions. Specifically, these explorations will provide a theoretical basis for selecting appropriate methods to inhibit or remove the contaminating elements (^58^Co and ^60^Co).

Austenitic stainless steel (SS) is commonly used in the chemical industry, construction, food production, and nuclear power fields due to its excellent mechanical properties and corrosion resistance [6]. In the primary loop system of a PWR nuclear power plant, 304SS is the main structural material of main pipes and core components [7]. To better understand the deposition behavior of cobalt on the surfaces of 304SS within the primary loop system of PWRs, it is crucial to understand the behavior of the radionuclides (^58^Co and ^60^Co) migrating to the surface of the steel and chemically interacting with related substances. Holdsworth et al. [5] studied the depth distributions of cobalt deposition on 316 stainless steel by simulating normal water conditions (NWC) and hydrogen water conditions (HWC) for a boiling water reactor. They found that cobalt was deposited on the surface of 316 stainless steel after 500 h. The maximum concentrations of cobalt on the metal surface under NWC (1.7 wt.%) and HWC (1.6 wt.%) were essentially the same, and the highest concentrations were found at the interface between the inner and outer oxide layers. However, the deposition depth of cobalt in the normal water environment (700 nm) was significantly greater than that in the hydrogen water environment (90 nm). Cobalt depth distributions were also reported in the study by Hosokawa et al. [8] and they generally agreed with those of the above study. In addition, Fuse et al. [9] studied radioactive contamination by cobalt by simulating the water environment of a boiling water reactor based on the Gibbs free energies of the reactions between metal ions and oxides; the cobalt on the surfaces of carbon and stainless steel was mainly deposited as CoFe_2_O_4_ and CoCr_2_O_4_, and there was a direct link between the concentration of cobalt and the formation of oxide films on the metal surface. Cantatore et al. [4] explained the formation of two cobalt contamination layers on a metal surface by using 1st principles modeling, and the addition of zinc to the solution inhibited the deposition of cobalt on the metal surface. Many experimental or simulation studies have been conducted for boiling water reactors, but there are not enough studies of cobalt deposition in the water cooling circuits of PWRs.

The main objective of this paper was to use SEM, XRD, LRS, XPS, GD-OES, and ICP-MS to investigate the microstructural characteristics and behavior of cobalt deposited on 304SS after immersion in 335 °C water containing 2.2 ppm LiOH, 1200 ppm H_3_BO_3_, and 500 ppm Co(NO_3_)_2_ for 240 h.

## 2. Experimental

### 2.1. Materials and Specimens

The chemical composition of the 304SS is listed in Table 1. The composition was determined by spark discharge atomic emission spectrometry (Spark-AES). The test specimens were cut to sizes of 10 mm × 10 mm × 2 mm. The 10 mm × 10 mm surface was ground with SiC papers of 180 to 2000 particle size. The specimens were then carefully cleaned in ethanol and acetone to remove surface contaminants. The XRD analysis showed that the 304SS consisted of a martensitic Fe (α) phase and an austenite Fe (γ) phase. The average grain size was 15.9 μm.

### 2.2. Experimental Procedures

The specimens were immersed in a static autoclave with a volume of 500 mL and maintained at 335 °C and 15.5 MPa for 240 h. The 250 mL solution in the autoclave contained 2.2 ppm LiOH, 1200 ppm H_3_BO_3_, and 500 ppm Co(NO_3_)_2_·6H_2_O. Under normal conditions, the saturated vapor pressure was 13.7 MPa in the closed environment at 335 °C. Before warming up, a booster pump was used to add 2.5 MPa of air to ensure that the pressure in the autoclave was 15.5 MPa when the temperature was raised to 335 °C. In the experiments, ^59^Co was chosen instead of the radionuclide ^60^Co because it has the same chemical properties as ^60^Co but is not radioactive, which facilitated safe handling and subsequent microstructural characterization.

After immersion, the specimens were dried. The surface morphologies of the specimens were examined by scanning electron microscopy (TESCAN CLARA SEM, Hitachi, Tokyo, Japan). The phases of the specimens were determined by X-ray diffraction (SmartLab SE XRD, Rigaku, Bruker AXS, Karlsruhe, Germany) with Cu Kα radiation. The compositions of the specimens’ surfaces after immersion were analyzed by laser Raman spectroscopy (LRS, Renishaw, Gloucestershire, Britain), using a selected band of 532 nm and a shift range of 150–800 cm^−1^. The chemical compositions of the specimens were investigated by X-ray photoelectron spectroscopy (AXIS SUPRA+ XPS, Thermo Fisher Scientific, Waltham, MA, USA). The photoelectrons were excited by a monochromatic Al Ka source operated at 15 kV with an initial photoenergy of 1486.6 eV. The resulting XPS spectra were calibrated with the standard carbon C 1s binding energy of 284.8 eV. The elemental depth profiles were analyzed using a GDA 750HP glow discharge optical emission spectrometer (GD-OES, Thermo Fisher Scientific, Waltham, MA, USA) with low detection limits (0.1–10 ppm) and a high depth resolution (~1 nm). In addition, GD-OES was applied to determine the depth distributions for elements in the nano-oxide films on the surface of 304SS [2,10]. The ion concentrations of the solution in the autoclave were measured by inductively coupled plasma emission mass spectrometry (ICP-MS, Thermo Fisher Scientific, Waltham, MA, USA) before and after 240 h of immersion.

## 3. Results

**Figure 1:**
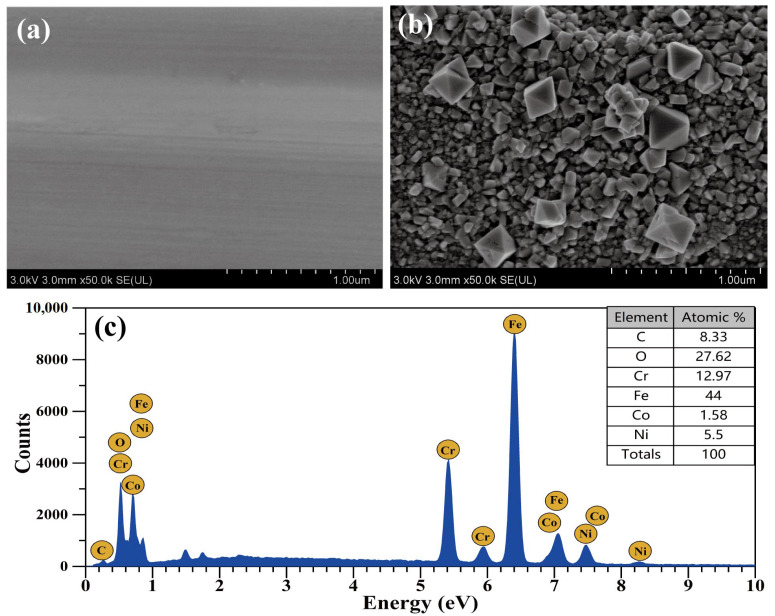
Surface morphology of the 304SS specimen at (**a**) 0 h and (**b**) 240 h. (**c**) Surface EDS spectrum of the 304SS specimen after 240 h.

Figure 1 shows the surface morphology and surface element distribution of the 304SS specimen before and after 240 h of immersion in high-temperature and high-pressure water containing 2.2 ppm LiOH, 1200 ppm H_3_BO_3_, and 500 ppm Co(NO_3_)_2_. From Figure 1a,b, it is obvious that only the scratches left by grinding were clearly visible on the surface of the original specimen; after 240 h of immersion, inconsistently sized particles appeared on the surface of the 304SS, and these particles completely covered the original scratches. Large particles were loosely arranged on the surface, the maximum diameter of which was approximately 400 nm. Small particles were closely arranged on the inner surface, and their size was approximately 50 nm. Medium-sized particles seemed to grow out of the small particles, and all particles exhibited spinel shapes. Similar spinel particles were identified in many previous experiments involving high-temperature and high-pressure oxidation of 304SS [11,12,13,14,15,16,17]. Figure 1c shows the EDS results for the surface particles shown in Figure 1b, which clearly indicated that the surface particles contained Co and O. Since EDS may also have detected elements contained in the 304SS substrate, it could only be assumed that Fe, Cr, and Ni may be present in the particles.

**Figure 2:**
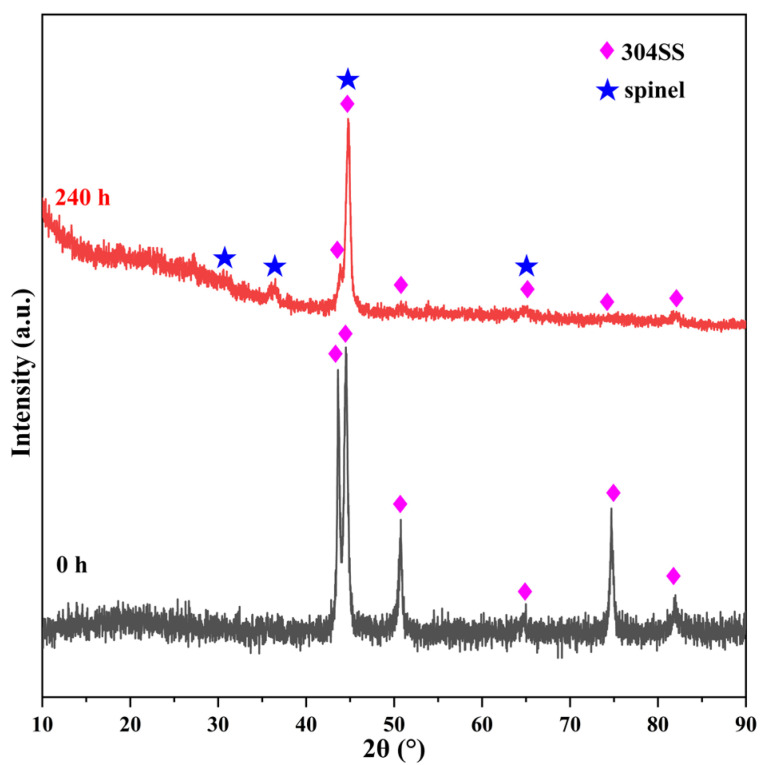
Surface XRD patterns for the 304SS specimen immersed in 335 °C water for 0 and 240 h.

Figure 2 shows the XRD patterns of the 304SS surface before and after 240 h of immersion. Before immersion, the six peaks at 43.5°, 44.5°, 64.7°, 50.8°, 74.7°, and 81.9° corresponded to the austenitic matrix phase (γ phase) and a small amount of residual martensitic phase (α phase) in the 304SS. After 240 h, two new weak peaks appeared at 30.3° and 35.7°, which coincided with the characteristic spinel peaks of MFe_2_O_4_ and MCr_2_O_4_ (M=Co, Fe, Ni). After 240 h, the XRD pattern showed spinel and 304SS substrate peaks, which were weakened compared to those of the original specimen. These results showed that the spinel particles formed on the surface of the 304SS were thin enough to allow the X-rays to penetrate through the layers and reach the 304SS substrate.

**Figure 3:**
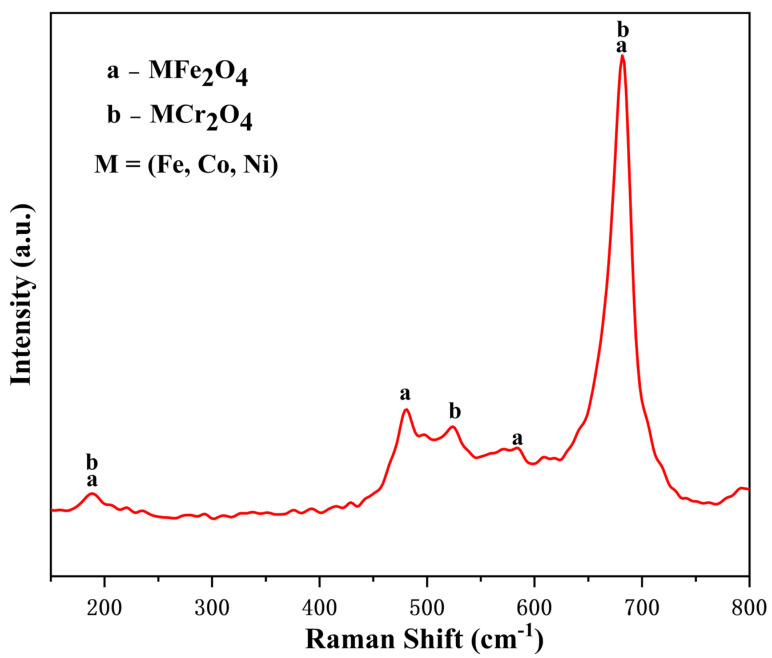
Surface Raman spectra of the 304SS specimen immersed in 335°C water for 240 h.

Figure 3 shows the Raman spectra of the surface particles formed on the 304SS after 240 h of immersion. The Raman peaks at 188, 481, 523, 571, and 681 cm^−1^ were those of the spinels MFe_2_O_4_ and MCr_2_O_4_ (M = Co, Fe, Ni), as previously reported [18,19,20,21]. The Raman spectrum of hematite was not observed in this study, in sharp contrast to the immersion of 304SS under high temperature and high pressure in a cobalt-free environment [22,23]. These results indicated that the surface of the 304SS comprised spinels, which was consistent with the XRD analysis.

**Figure 4:**
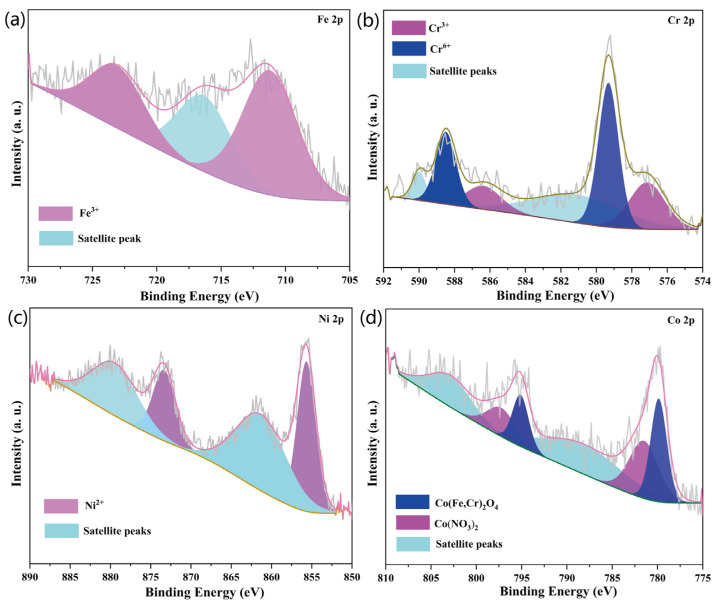
Surface XPS spectra of the 304SS specimen immersed in 335 °C water for 240 h. (**a**) Fe 2p core level spectra, (**b**) Cr 2p core level spectra, (**c**) Ni 2p core level spectra, and (**d**) Co 2p core level spectra.

The Fe 2p, Cr 2p, Ni 2p, and Co 2p XPS spectra determined for the surface of the 304SS after immersion in a 335 °C solution containing 2.2 ppm LiOH, 1200 ppm H_3_BO_3_, and 500 ppm Co(NO_3_)_2_ for 240 h are shown in Figure 4. Two major Fe 2p peaks are present in Figure 4a; the main peaks at about 723.1 eV (Fe 2p1/2) and 711.2 eV (Fe 2p3/2) were assigned to Fe^3+^ [24] and indicated the possible presence of Fe_2_O_3_, CoFe_2_O_4_, and NiFe_2_O_4_ [25,26,27]. Figure 4b shows three pairs of Cr 2p XPS peaks; the peaks at 576.8 and 586.3 eV were assigned to Cr^3+^ ions in Cr_2_O_3_, NiCr_2_O_4_, FeCr_2_O_4_, and CoCr_2_O_4_ [28,29,30]. The Cr 2p_3/2_ binding energy of 579.3 eV and the Cr 2p_1/2_ binding energy of 588.40 eV were attributed to Cr^6+^ [28], which was produced by the oxidation of Cr^3+^. The Cr 2p peaks at 581.1 and 589.9 eV were characteristic satellite peaks [31]. Two pairs of Ni 2p peaks are seen in Figure 4c; the Ni 2p_3/2_ binding energy of 855.6 eV and the Ni 2p_1/2_ binding energy of 873.3 eV were attributed to Ni^2+^ in NiO, NiCr_2_O_4_, and NiFe_2_O_4_ [26,32,33]. The Ni 2p peaks at 861.3 and 879.4 eV were satellite peaks [32,34]. Three pairs of Co 2p peaks are seen in Figure 4d; among them, the peaks at 780.1 and 795.1 eV were attributed to CoCr_2_O_4_ and CoFe_2_O_4_, respectively [33,35,36]. The peaks at 781.5 and 797.2 eV were assigned to Co(NO_3_)_2_ [37]. The Co 2p peaks at 788.1 and 802.8 eV were satellite peaks [38].

**Figure 5:**
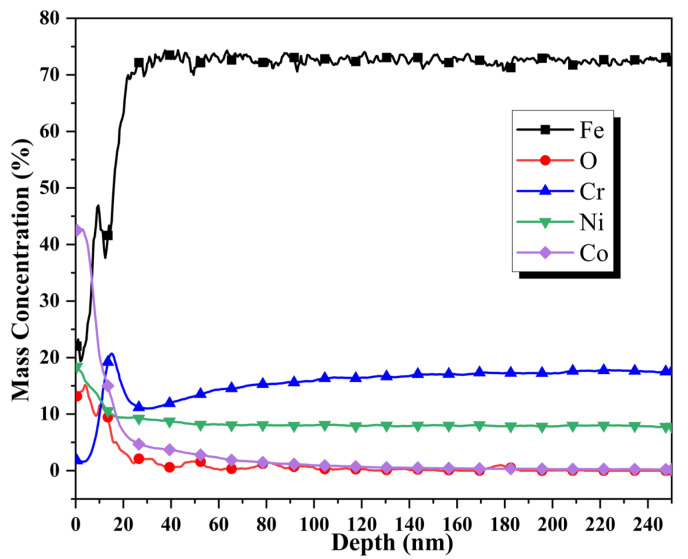
Elemental depth profiles of the surface particle layer formed on the 304SS specimen immersed in 335 °C water for 240 h.

Figure 5 shows the depth distributions of the elemental contents of Fe, O, Cr, Ni, and Co on the surface of the 304SS after 240 h of immersion in a 335°C solution containing 2.2 ppm LiOH, 1200 ppm H_3_BO_3_, and 500 ppm Co(NO_3_)_2_. The depth distributions were examined by GDOES. From the outer to the inner layers of the particles, the Co content gradually decreased from 42.7% to 2%, the same content as the substrate, over a cobalt diffusion depth of approximately 200 nm. The Fe content gradually increased from 22% to 46.9% at 10 nm, then decreased to 38%, which corresponded to a depth of 16 nm, and then increased again, reaching the substrate content (72%) at 26 nm. Similarly, the Cr content gradually increased from 1.7%, reaching a maximum peak value of 20% at 16 nm, and then gradually decreased to the level of the substrate. It is noteworthy that both the Ni and O contents gradually decreased from levels higher than the substrate content down to the substrate content, although the Ni diffusion depth was smaller than that of O, and the maximum O diffusion depth remained almost the same as that of Co.

**Figure 6:**
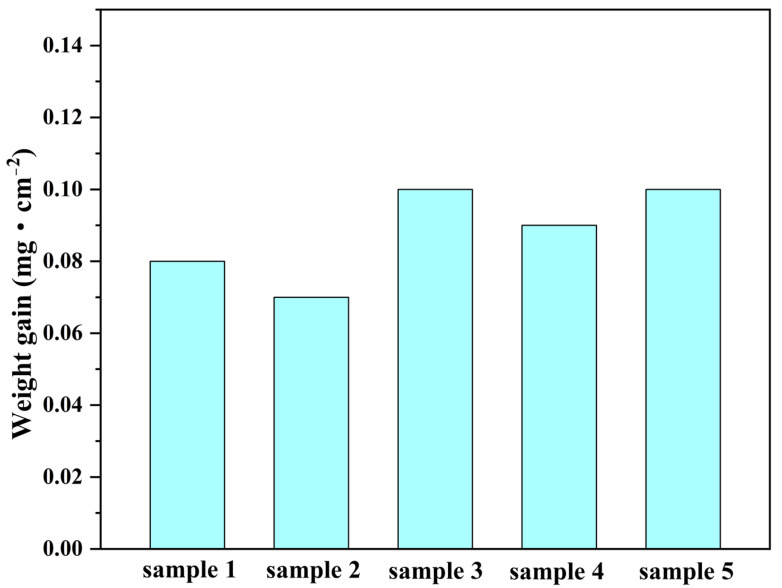
Diagram showing the weight gains of the 304SS specimen immersed in 335 °C water after 240 h.

Figure 6 shows the weight gain of the 304SS after immersion in a 335 °C solution containing 2.2 ppm LiOH, 1200 ppm H_3_BO_3_, and 500 ppm Co(NO_3_)_2_ for 240 h. Five parallel specimens were tested to reduce the error. The results showed that the average weight gain of the five specimens was 0.088 mg·cm^−2^ after 240 h of immersion, and the weight changes were very small.

Table 2 shows the Fe/Cr/Ni/Co ion concentrations in the solution before and after immersion for 240 h. After 240 h, only the Co ion concentration in the solution showed a significant decrease, from 101.1 to 1.09 mg/L. In contrast, the Fe/Cr/Ni ion concentrations increased. The Ni ion concentration rose from 0.001 to 104.32 mg/L, and the Cr ion concentration rose from 0.001 to 82.02 mg/L. The Fe ion concentration increased slowly, from 0.001 to 0.04 mg/L.

## 4. Discussion

According to the SEM and EDS results (Figure 1), cobalt was deposited on the 304SS in the form of two-layer spinels. The outer layer was composed of relatively large and loose spinel particles, while the inner layer was composed of relatively small and compact spinel particles. The particles on the 304SS exhibited the spinel structures of Co(Fe, Cr)_2_O_4_, but not CoO, Co_2_O_3_, Co(OH)_2_, or CoOOH [39,40,41,42]. In the studies by Hetache et al. [43] and Ebrahimifar [44], it was reported that the CoFe_2_O_4_ and CoCr_2_O_4_ particles exhibited spinel shapes. Hence, it was possible to use their morphologies to identify the phase structures of the particles in this study. The XRD patterns (Figure 2) and Raman spectra (Figure 3) of the current study showed that the particles formed on the 304SS immersed in a 335 °C solution containing 2.2 ppm LiOH, 1200 ppm H_3_BO_3_, and 500 ppm Co(NO_3_)_2_ for 240 h mainly contained spinel phases. The XRD standard peaks at 30.3°, 35.7°, 44.5°, and 64.7° corresponded completely with Co(Fe, Cr)_2_O_4_ [45,46]. Similarly, the Raman peaks at 188, 481, 523, 571, and 681 cm^−1^ also corresponded with Co(Fe, Cr)_2_O_4_ [18,19]. The Fe, Co, and Cr XPS spectra (Figure 4a,b,d) indicated that the metallic elements were Fe^3+^, Co^2+^, Cr^3+^, and Cr^6+^ [26,28,29,33]. Since the particle layer only contained the spinel phase, it could not contain hematite iron or chromium. Thus, it was confirmed that the cobalt on the surface of the 304SS was contained in CoFe_2_O_4_ and CoCr_2_O_4_ after 240 h of high-temperature water immersion.

According to the GDOES results (Figure 5), the contents of cobalt and oxygen gradually decreased from depths of 0 to 200 nm, indicating that cobalt ferrite and cobalt chromate were distributed throughout the entire particle layer. Although the iron content for the entire particle layer was lower than that in the substrate, a peak for iron appeared at a depth of 10 nm. This indicated that during the high-temperature water immersion process, the iron from the 304SS dissolved into the solution and then reprecipitated on the 304SS. The GDOES results showed that after 240 h of immersion, the cobalt and oxygen contents on the 304SS had significantly increased. However, the weight data for the 304SS (Figure 6) showed that the weights of the samples remained almost unchanged after immersion, indicating that metals from the 304SS had dissolved. In Table 2, the increased chromium and nickel ion contents also indicated that metals from the 304SS had dissolved. Previous work showed that the amounts of 304SS surface metals that dissolved in the hot water decreased in the order Fe > Ni > Cr [30,47,48]. However, the concentration of iron ions in the solution was very low (Table 2), which indicated that some iron ions had precipitated with the cobalt ions to form CoFe_2_O_4_. The published literature on high-temperature water oxidation of 304SS has shown that the outer layer of NiFe_2_O_4_ is precisely produced through a dissolution–precipitation mechanism without cobalt participation [23,49]. Therefore, it can be inferred that CoFe_2_O_4_ mainly formed in the outer layer. Unlike the depth distribution of the iron content, the chromium content showed a peak value higher than that of the 304SS substrate at a depth of 16 nm. Therefore, it was inferred that CoCr_2_O_4_ was mainly formed in the inner particle layer. The chromium content and the particle distributions of the inner layer indicated that CoCr_2_O_4_ may have been formed through a solid-state reaction and ion exchange mechanism. According to research by Kuang et al. [23,49], the solid state reaction of Cr results in the formation of NiCr_2_O_4_ and FeCr_2_O_4_ in the inner layer of the oxide film following the high-temperature and high-pressure water oxidation of 304SS. When cobalt ions are present, Co competes with Ni and Fe in the crystal structures of NiCr_2_O_4_ and FeCr_2_O_4_, in which Ni and Fe occupy the tetrahedral positions. Ni and Fe can be substituted by Co to create CoCr_2_O_4_ [50]. Therefore, the depth distributions of the elements indicated that after 240 h of immersion, the cobalt deposition layer on the 304SS comprised an outer layer of CoFe_2_O_4_ and an inner layer of CoCr_2_O_4_. The outer and inner layers exhibited nanoscale thicknesses.

**Figure 7:**
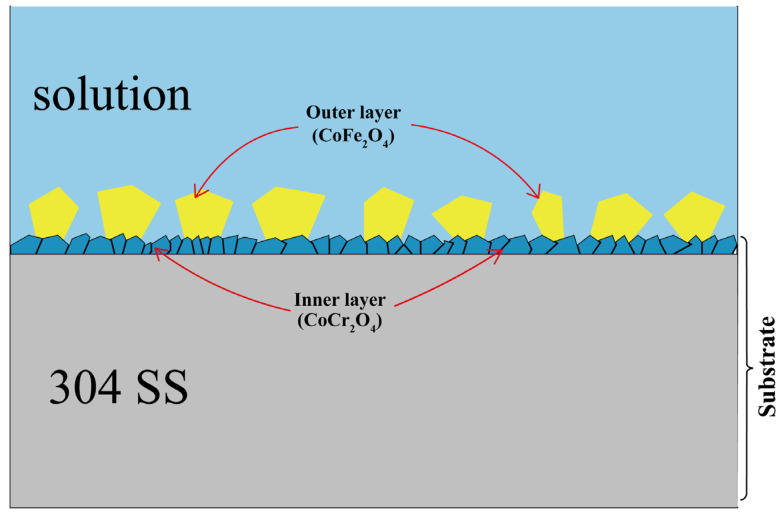
Schematic drawing of the cobalt particle layer formed on 304SS immersed in a 335 °C solution containing 2.2 ppm LiOH, 1200 ppm H_3_BO_3_, and 500 ppm Co(NO_3_)_2_ for 240 h.

Figure 7 shows a schematic structure of the cobalt deposition on the 304SS immersed in high-temperature water in this study. First, due to preferential dissolution of the iron and nickel from 304SS, CoCr_2_O_4_ was formed on the metal surface through solid-state reactions and ion exchange. Additionally, the iron and cobalt ions from the solution formed CoFe_2_O_4_ on the upper layer of CoCr_2_O_4_ by coprecipitation.

## 5. Conclusions

In this study, cobalt deposition on 304SS immersed for 240 h in a 335 °C solution containing 2.2 ppm LiOH, 1200 ppm H_3_BO_3_, and 500 ppm Co(NO_3_)_2_ was investigated with SEM, XRD, LRS, XPS, GDOES, and ICP-MS. The conclusions are summarized as follows:

1. After immersion for 240 h, the cobalt ions from the solution were deposited on the 304SS in the form of spinels CoFe_2_O_4_ and CoCr_2_O_4_, with deposition depths of approximately 200 nm.
2. Similar to the oxide film structure of 304SS after high-temperature water oxidation, after 240 h of immersion, two cobalt deposition layers were formed on the surface of the 304SS. Dense and fine spinel particles of CoCr_2_O_4_ were deposited on the inner layer, and loose, large spinel particles of CoFe_2_O_4_ were deposited on the outer layer.
3. The outer layer of CoFe_2_O_4_ was mainly formed through a dissolution–coprecipitation mechanism, while the inner layer of CoCr_2_O_4_ was formed through a solid-state reaction and ion exchange mechanism.

These three points reveal the behavior of cobalt deposited on 304SS in borated and lithiated high-temperature water, and the results have a certain reference value for exploring the deposition behavior and mechanism of radionuclide cobalt on 304SS in the PWR primary loop water environment.

## Figures and Tables

**Table 1:** Chemical composition of the 304SS used.

Elements	C	Si	Mn	S	P	Co	Ni	Cr	Fe
Amounts (wt.%)	0.05	0.45	0.95	0.005	0.036	0.23	7.93	18.2	Bal.

**Table 2:** ICP-MS data showing the Fe/Cr/Ni/Co concentrations in solution at 0 h and after 240 h (mg/L).

Elements		Fe	Cr	Ni	Co
Time (h)	0	0.001	0.001	0.001	101.1
	240	0.04	82.02	104.32	1.09

## Data Availability

Not applicable.
